# CT-guided hook-wire localization of malignant pulmonary nodules for video assisted thoracoscopic surgery

**DOI:** 10.1186/s13019-020-01279-9

**Published:** 2020-10-09

**Authors:** Huijun Zhang, Ying Li, Nadier Yimin, Zelai He, Xiaofeng Chen

**Affiliations:** 1 Department of Cardiothoracic Surgery, Huashan Hospital of Fudan University, Shanghai, 200040 China; 2 Department of Thoracic Surgery, Shanghai Pulmonary Hospital, Tongji University School of Medicine, Shanghai, China; 3 Department of Radiation Oncology, The First Affiliated Hospital of Bengbu Medical College, Bengbu, 233000 China

**Keywords:** Malignant pulmonary nodules, Hook-wire localization, Video assisted thoracoscopic surgery

## Abstract

**Objectives:**

Video assisted thoracoscopic surgery (VATS) can currently be used to diagnose and treat pulmonary nodules. However, intraoperative location of pulmonary nodules in VATS is challenging due to their small diameter and deep location in the pulmonary parenchyma. The purpose of this study was to report the clinical safety and effectiveness of CT-guided hook-wire for preoperative localization of malignant pulmonary nodules smaller than 1 cm in diameter.

**Methods:**

From February 2017 to January 2018, we collected the data of 80 patients with malignant pulmonary nodules less than 1 cm in diameter who underwent CT-guided hook-wire preoperative localization and VATS surgery. The effectiveness of preoperative localization was evaluated based on surgical duration, success rate of VATS surgery, and localization-related complications.

**Results:**

The diameter of pulmonary nodules were 0.85 ± 0.17 mm with a distance to the pleural surface of 19.66 ± 14.10 mm. The length of the hook-wire in the lung parenchyma was 29.17 ± 13.14 mm and hook-wire dislodgement occurred in 2 patients. Complications included 27 cases of minor pneumothorax and 18 cases of mild parenchymal hemorrhage. A significant correlation was observed between the length of the hook-wire in the lung parenchyma and mild parenchymal hemorrhage (*P* = 0.044). The average time of hook-wire localization was 9.0 ± 2.6 min and the average operation time for VATS was 89.02 ± 23.35 min without conversion thoracotomy.

**Conclusions:**

CT-guided hook-wire localization of the lesion during VATS resection is safe for malignant pulmonary nodules with diameter less than 1 cm.

## Introduction

Low-dose spiral computed tomography (CT) screening for early lung cancer is a promising strategy for improving lung cancer survival and can reduce lung cancer deaths by 20% [1]. Due to the widespread use of low-dose spiral CT, an increasing number of pulmonary nodules have been detected in the early stages of lung cancer, especially those featuring ground-glass opacity (GGO). Of these nodules, 59–73% are malignant, therefore rapid and accurate histological diagnosis and treatment are necessary [2]. The effectiveness of routine procedures such as transbronchial or CT-guided fine-needle aspiration biopsy may be limited by the small diameter of the pulmonary nodule, making it difficult to locate accurately. When less invasive techniques are not available for accurate diagnosis, resection of the pulmonary nodule with video assisted thoracoscopic surgery (VATS) is often recommended for definitive diagnosis and treatment. VATS is widely used for the treatment of pulmonary nodules and provides less discomfort and reduced postoperative complications compared to standard thoracotomy [3, 4]. However, most pulmonary nodules (especially with GGO) are inaccessible by finger palpation during VATS, which may lead to an increase in thoracotomy up to 46% [5, 6]. Therefore, the localization of pulmonary nodules during VATS is an important ongoing issue.

Several techniques exist for pulmonary nodule localization including finger palpation [7], intraoperative ultrasound [8], hook-wire [9], micro-coil [10], and methylene blue [11]. The detection rate of Lipiodol [12] or radionuclide [13] is as high as 100% [14]. Pulmonary nodule localization-related complications are usually asymptomatic pneumothorax or mild parenchymal hemorrhage and more severe complications are rarely reported [15, 16]. In this study, we report the safety and effectiveness of preoperative CT-guided hook-wire for the localization of malignant pulmonary nodules smaller than 1 cm in diameter.

## Methods

### CT-guided hook-wire localization

**Fig. 1:**
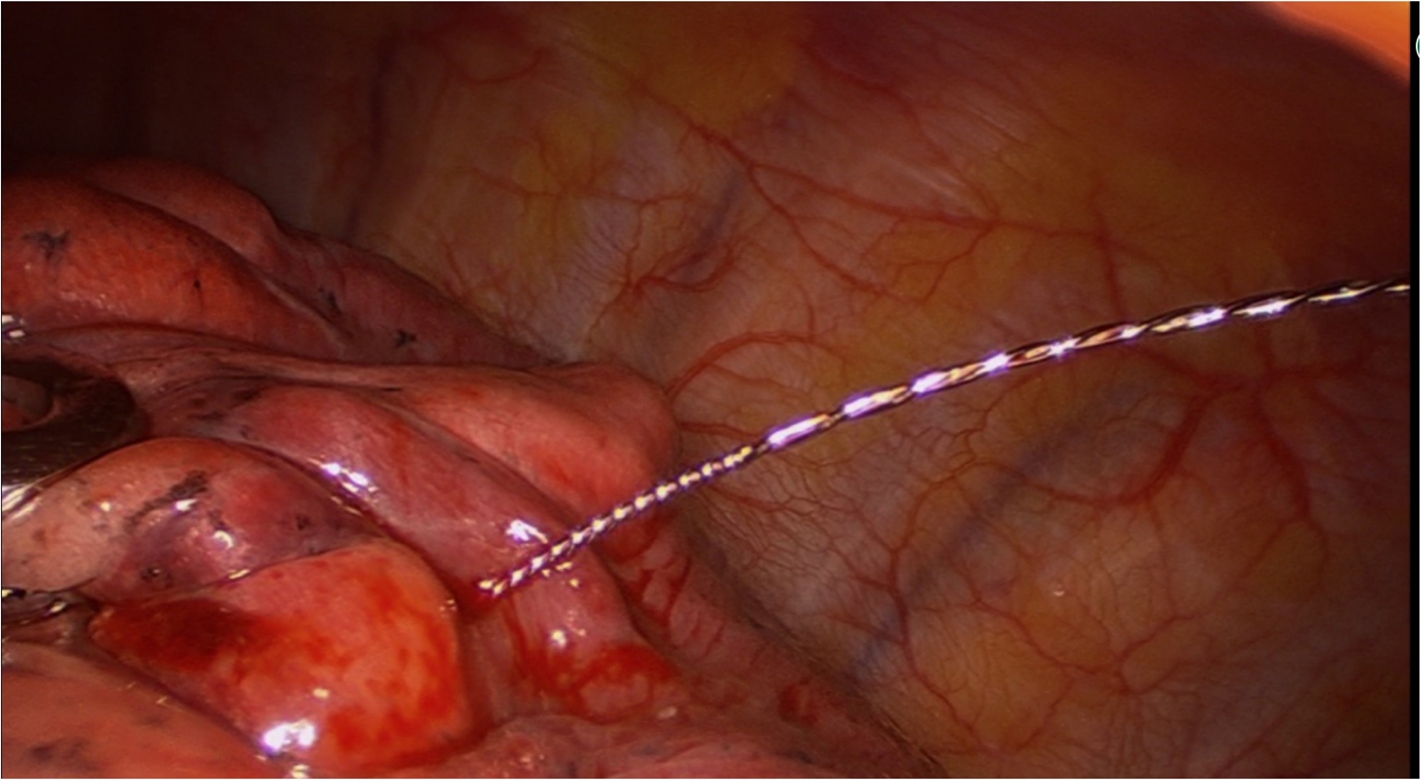
The hook-wire needle was inserted into the lung parenchyma

**Fig. 2:**
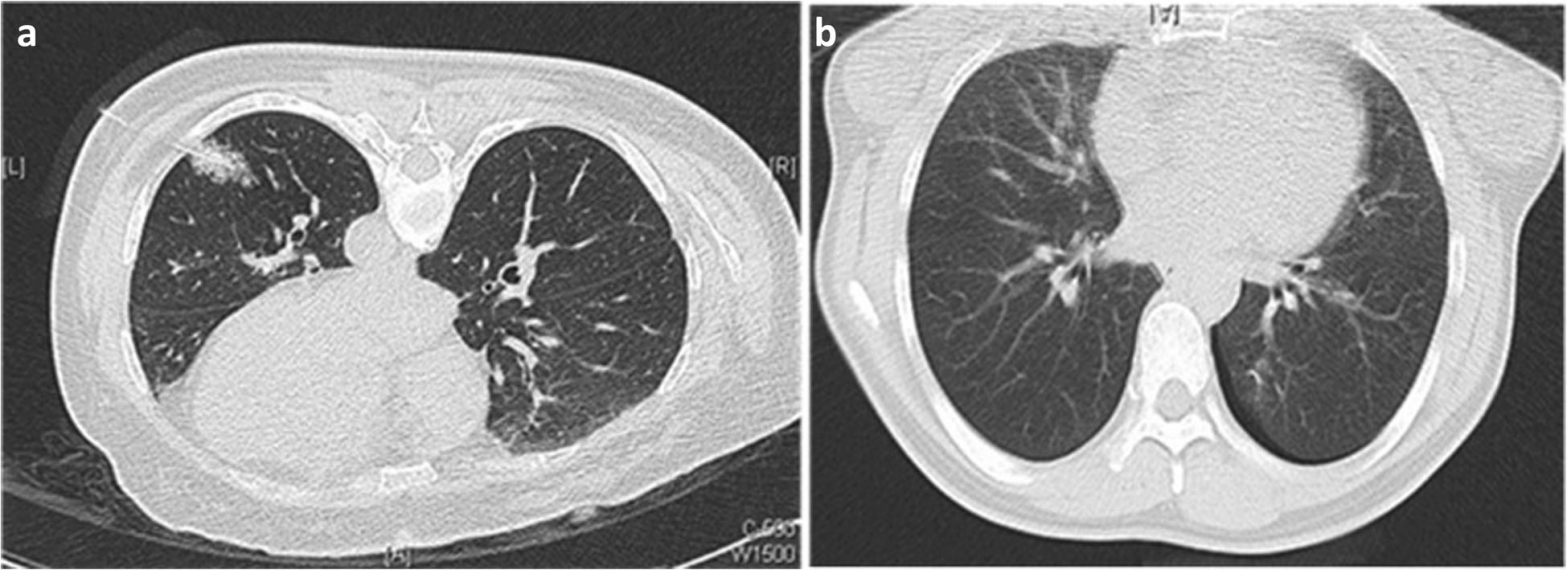
Mild pulmonary hemorrhage was observed in a 41-year man **a**; Minor pneumothorax was observed in a 51-year woman **b**

A retrospective analysis was performed on 80 patients from February 2017 to January 2018 who underwent VATS resection after receiving preoperative CT-guided hook-wire localization of malignant pulmonary nodules less than 1 cm in diameter. The double-barbed wire system (hook-wire localization needles) consisted of a 20-gauge breast puncture needle 10.7 cm long and a BARD DUALOK trocar (Bard Peripheral Vascular, USA). First, a low-dose CT scan with a section thickness of 1.5 mm was performed to confirm the accurate localization of pulmonary nodules. The puncture needle was placed at the shortest distance between the skin and the pulmonary nodules while avoiding occlusion of the ribs and scapula. After the location of the puncture was determined, we locally anaesthetised the patient. With the guidance of CT, the double-barbed wire system was gradually inserted into the lung parenchyma and the tip of the double barb was expanded as close to the nodules as possible or through the nodules themselves (Fig. 1). After successful localization, a CT scan was performed to further confirm the exact location of the hook and the presence of complications such as pneumothorax or parenchymal hemorrhage (Fig. 2). After the device was removed, the portion of the needle outside the patient’s body was cut off. Patients should avoid physical activity after hook-wire localization and be transferred directly to the operating room.

### VATS

**Fig. 3:**
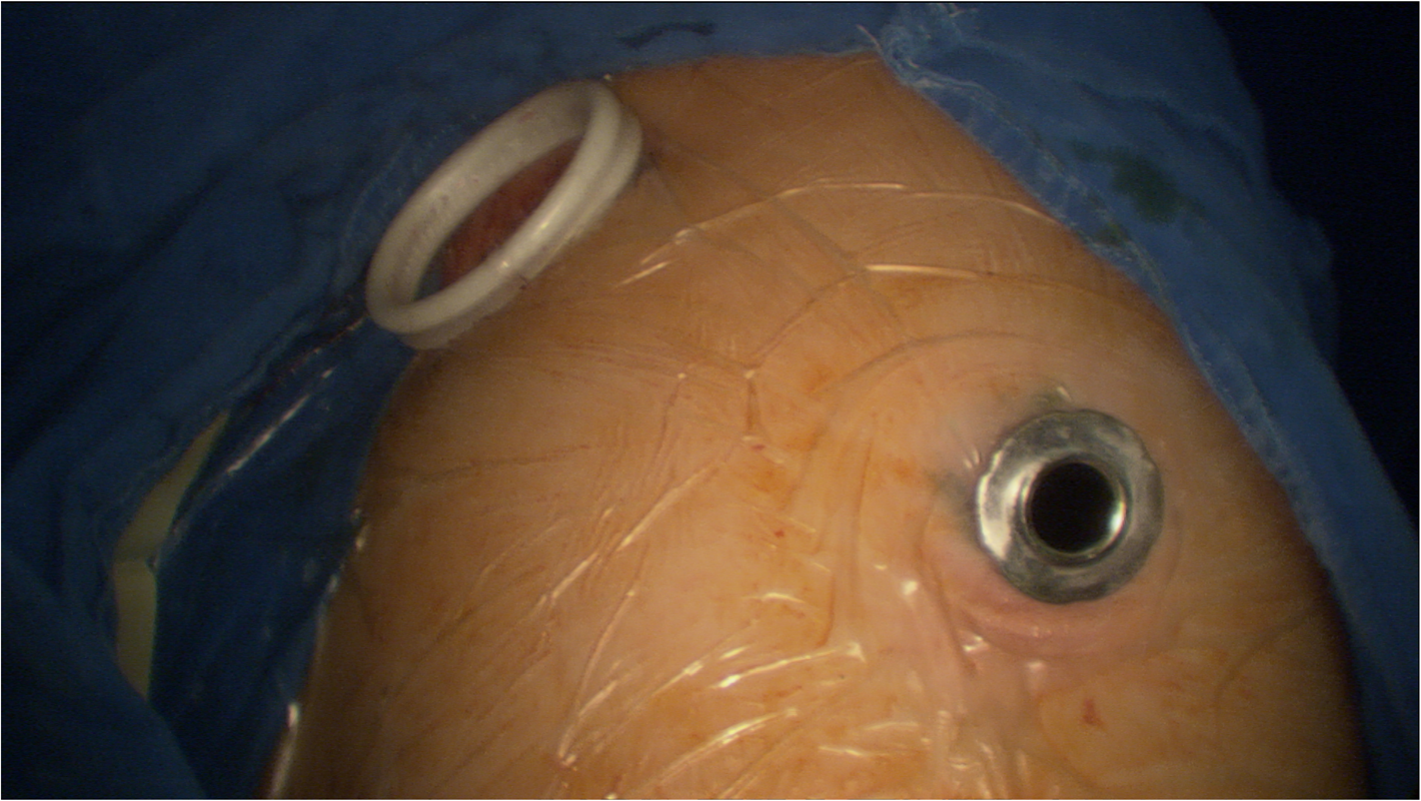
VATS resection was performed using a two-port approach

Patients undergoing VATS were placed under general anaesthesia. VATS resection includes lung lobectomy, segmentectomy, or wedge resection. The thoracic surgeon and the radiologist discussed each case before VATS surgery. The intubation included a double-lumen endobronchial tube and immediate one-lung ventilation to avoid the potential risk of puncturing the lungs with positive pressure and triggering tension pneumothorax. Surgical resection was performed using a two-port approach with the camera placed in the seventh intercostal space on the midaxillary line, The operating incision was placed in the fourth intercostal space on anterior axillary line (Fig. 3). After entering the chest cavity, the positioning needle and the pleura on the lung surface were fixed with homelock or suture to avoid needle movement. All nodules were extracted in protective bags to prevent the chest wall from being embedded with malignant cells. The chest cavity was flushed with saline solution and a chest tube with drainage system was placed.

### Statistical analysis

All statistical analyses were performed using SPSS software. Descriptive statistics of continuous variables were expressed as mean values ± SD. Bivariate correlation analysis was performed using Pearson correlation analysis and *P* < 0.05 was considered statistically significant.

## Results

Our 80-patient cohort included 24 male and 56 females between 27 and 82 years old (50.65 ± 11.75). The diameter of malignant pulmonary nodules was 0.85 ± 0.17 cm (0.3–1.0 cm). We located the pulmonary nodules in the right upper lobe in 32 cases (40%), the middle lobe in 8 cases (10%), the right lower lobe in 18 cases (22.5%), the left upper lobe in 15 cases (18.7%), and the left lower lobe in 8 cases (10%). The distance between the pulmonary nodules and the pleural surface was 19.66 ± 14.10 mm (0–85 mm), but the length of the localization needle in the lung parenchyma averaged 29.17 ± 13.14 mm (10–80 mm). Hook-wire dislodgement occurred in 2 patients (2.5%). The average time of hook-wire localization under CT guidance was 9.0 ± 2.6 min (5–18 min). The mean operation time of VATS was 89.02 ± 23.35 min (20–120 min) and there was no conversion to thoracotomy. During VATS, the location of pulmonary nodules was successfully found by tracing the needle on the puncture site of the lung surface. CT scan showed minor pneumothorax in 27 patients (33.7%). These patients had no obvious symptoms of chest tightness, shortness of breath, and other discomforts, so thoracic drainage was not required. Mild parenchymal hemorrhage developed in 18 patients (22.5%) with no serious clinical consequences (Fig. 1)(Table 1). We did not observe any haemothorax or pulmonary embolism. The length of hook-wire in the lung parenchyma did not correlate with the occurrence of minor pneumothorax, but was significantly associated with the occurrence of mild pulmonary parenchymal hemorrhage (*P* = 0.044). In additionally, the occurrence of minor pneumothorax and mild pulmonary hemorrhage was not affected by the depth of pulmonary nodules (Table 2). After hook-wire localization, all lesions were successfully removed by VATS including 10 cases of lobectomy (12.5%), 66 cases of segmentectomy (82.5%), and 4 cases of wedge resection (0.05%). CT features of malignant pulmonary nodules showed that 54 patients (67.5%) had pure GGO nodules and the remaining 26 patients (32.5%) had partially solid GGO nodules. Pathological examination revealed 10 cases of adenocarcinoma in situ (AIS,12.4%), 51 cases of micro-invasive adenocarcinoma (MIA, 63.8%), and 19 cases of invasive adenocarcinoma (stage IA, 23.8%) (Table 1).

**Table 1:** Patient and nodule characteristics

Characteristics	Value
Gender (Male/Female)	24/56
Age (years)	50.65 ± 11.75 (27–82)
Smoking history	14 (17.5%)
CT nodule size (mm)	0.85 ± 0.1 (3–10)
Nodule distance to pleural surface (mm)	19.66 ± 14.10 (0–85)
Length of hook-wire in lung parenchyma (mm)	29.17 ± 13.14 (10–80.0)
Nodule location	
RUL	32 (40%)
RML	8 (10%)
RLL	18 (22.5%)
LUL	15 (18.7%)
LLL	8 (10%)
Nodule feature	
pGGN	54 (67.5%)
mGGN	26 (32.5%)
Duration of hook wire placement (min)	9.0 ± 2.6 (5–18)
Duration of VATS procedure (min)	89.02 ± 23.35 (20–120)
Complication of hook wire	
Minor pneumothorax	27 (33.7%)
Mild pulmonary hemorrhage	18 (22.5%)
Hookwire dislodgement	2 (2.5%)
Histopathologic results	
AIS	10 (12.4%)
MIA	51 (63.8%)
IA	19 (23.8%)
Surgical type	
Wedge	4 (0.05%)
Segmentectomy	66 (82.5%)
lobectomy	10 (12.5%)

**Table 2:** Results of Spearman correlation analysis

Variable		Minor pneumothorax	Mild pulmonary hemorrhage
Length of hook-wire in lung parenchyma	r_s_	0.126	0.226
	*P*-value	0.226	**0.044**
Nodule distance to pleural surface	r_s_	−0.078	0.189
	*P*-value	0.492	0.092

r_s_: Spearman’s rank correlation coefficient

## Discussion

As one of the most common cancers, lung cancer is the leading cause of cancer death worldwide and its mortality rate exceeds the sum of the three most common cancers (colon, breast, and pancreatic cancer). Due to the lack of clinical symptoms in the early stage, most lung cancers are diagnosed as distant metastasis and the 5-year survival is only 17.8% [17]. With the widespread use of low-dose spiral CT, more pulmonary nodules, especially those with GGO, are found in the early stages. The prevalence of pulmonary nodules detected on by CT is 31% and up to 50% among high-risk patients [18]. Currently, the diagnosis of pulmonary nodules also includes positron emission tomography (PET) with 18-fluorodeoxyglucose (FDG), percutaneous needle aspiration biopsy, and transbronchic needle biopsy. Since 18F-FDG-PET/CT has insufficient sensitivity and specificity for the diagnosis of malignant pulmonary nodules, it cannot replace the “gold standard” pathology method based on resection or percutaneous biopsy [19]. Percutaneous needle biopsy and transbronchial needle biopsy are also very useful procedures for the diagnosis of malignant pulmonary nodules. Although both are much less invasive than surgery, but malignancy could not be reliably ruled out due to insufficient tissue sample or failure of the biopsy [20]. Under the guidance of CT, the accuracy of the needle biopsy is 88%, the sensitivity is 90%, and the false negative rate is 22% [21, 22]. However, due to limitations of the technique, some pulmonary nodules cannot be clearly diagnosed. As a minimally invasive method for diagnosis and treatment, VATS has been widely used for pulmonary nodules. However, some pulmonary nodules cannot be seen by the naked eye and cannot be clearly palpated by fingers during the operation, bringing further challenges in clinical diagnosis and treatment [23]. Some studies have shown that intraoperative finger-assisted palpation of pulmonary nodules with diameter of > 2 cm on the pleural surface is often possible. However, for small lesions < 1 cm in diameter, preoperative localization is usually recommended [24]. In this retrospective study, all 80 patients with malignant pulmonary nodules < 1 cm in diameter were treated with hook-wire localization.

At present, a variety of preoperative localization methods have been reported. However, each localization method has its advantages and disadvantages [25]. It is difficult to identify pulmonary nodules by methylene blue because it can quickly spread to the pleural surface [26]. Specific radioactive tracers, such as 99 m Technetium, are feasible for preoperative localization of small pulmonary nodules [27]. Although specific radioactive tracers are more accurate for preoperative localization of superficial pulmonary nodules, specialised equipment such as CT-fluorescence is required [28], and surgeons and radiologists could be exposed to radiation. In addition, the localization of deep pulmonary nodules is blurred, which limits its clinical use. Intraoperative ultrasound detection requires a specific flexible ultrasound probe and the localization is impeded by air, therefore it is difficult to be applied in patients with non-collapsed lung or emphysema [29]. Currently, there is no golden standard for preoperative localization in clinical practice.

Hook-wire localization is widely used and has the advantages of simplicity and ease of operation, short operation time, and reduced surgical injury. Ciriaco et al. [30] report that the average time of CT-guided pulmonary nodules localization is 20 ± 10 min, which is slightly longer than the preoperative localization time in our study, which was similar to the duration reported by Li et al. [31]. Interestingly, Ciriaco et al. report that the VATS time of pulmonary nodules after preoperative localization was 40 ± 7 min compared with 75 ± 12 min without preoperative localization. This indicates that with the help of preoperative localization, pulmonary nodules are identified and removed faster during surgery, which greatly shortens the operation time. In comparison, the average operation time of VATS in our study was 89.02 ± 23.35 min. The discrepancy can be explained by the high proportion of lung segmentectomy and lobectomy in our study population (95%), which complicated the surgery and increased operation time.

Inevitably, the hook-wire preoperative localization also has some disadvantages. For example, the surgical margin cannot be determined after displacement of the localization needle and therefore the complete removal of pulmonary nodules cannot be guaranteed [32]. It has been reported that 3 to 8% of localization needles may be displaced or even detached [33], which was consistent with the occurrence in our study. The causes of hook-wire dislodgement were mostly by coughing and upper body movement. In this situation, we determined the location of pulmonary nodules by the puncture point of the hook-wire on the lung surface.

Pulmonary nodules that are deep in the lung or blocked by the ribs and scapula are difficult to locate, leading to pneumothorax, haemothorax, pain, and death from gas embolisms [34]. Huang et al. reported that in 39 patients undergoing preoperative localization, 5.2% of the patients presented mild parenchymal hemorrhage and 12.8% presented minor pneumothorax [15]. Other reports have described that the incidence of mild parenchymal hemorrhage is 13.9–36% and the incidence of pneumothorax is 7.5–40% [16]. In this study, minor pneumothorax and mild parenchymal hemorrhage occurred in 33.7 and 22.5% of the cases, respectively, which was consistent with previous studies. No serious complications such as massive haemothorax or massive air embolism resulted from these incidents. Interestingly, we found that the length of the localization needle in the pulmonary parenchyma had a significant correlation with the occurrence of mild pulmonary parenchymal hemorrhage, although the depth of pulmonary nodules did not show such correlations. Therefore, we suggest that physicians should be aware of the risk of pulmonary hemorrhage for localization of pulmonary nodules requiring a long needle.

This study has some limitations. Primarily, there was no control group of VATS patients without preoperative localization. This may lead to overestimation of the safety and effectiveness of preoperative hook-wire localization.

## Conclusion

CT-guided hook-wire preoperative localization of malignant pulmonary nodules with diameter < 1 cm is safe and effective before VATS resection.

## Data Availability

The datasets used and analysed during the current study are available from the corresponding author on reasonable request.

## Abbreviations

CT: Computed Tomography
GGO: Ground-Glass Opacity
VATS: Video-Assisted Thoracoscopic Surgery

## References

[1] Aberle DR, Adams AM, Berg CD, Black WC, Clapp JD, National Lung Screening Trial Research Team (2011). Reduced lung-Cancer mortality with low-dose computed tomographic screening. N Engl J Med.

[2] Gould MK, Donington J, Lynch WR, Mazzone PJ, Midthun DE, Naidich DP (2013). Evaluation of individuals with pulmonary nodules: when is it lung cancer? Diagnosis and management of lung cancer, 3rd ed: American College of Chest Physicians evidence-based clinical practice guidelines. Chest..

[3] Rivera MP, Mehta AC, Wahidi MM (2013). Establishing the diagnosis of lung cancer: diagnosis and management of lung cancer, 3rd ed: American College of Chest Physicians evidence-based clinical practice guidelines. Chest..

[4] Bernard A (1996). Resection of pulmonary nodules using video-assisted thoracic surgery. The thorax group. Ann Thorac Surg.

[5] Greenfield AL, Steiner RM, Liu JB, Cohn HE, Goldberg BB, Rawool NM (1997). Sonographic guidance for the localization of peripheral pulmonary nodules during thoracoscopy. AJR Am J Roentgenol.

[6] Santambrogio R, Montorsi M, Bianchi P, Mantovani A, Ghelma F, Mezzetti M (1999). Intraoperative ultrasound during thoracoscopic procedures for solitary pulmonary nodules. Ann Thorac Surg.

[7] Suzuki K, Nagai K, Yoshida J, Ohmatsu H, Takahashi K, Nishimura M (1999). Video-assisted thoracoscopic surgery for small indeterminate pulmonary nodules: indications for preoperative marking. Chest..

[8] Kondo R, Yoshida K, Hamanaka K, Hashizume M, Ushiyama T, Hyogotani A (2009). Intraoperative ultrasonographic localization of pulmonary ground-glass opacities. J Thorac Cardiovasc Surg.

[9] Ichinose J, Kohno T, Fujimori S, Harano T, Suzuki S (2013). Efficacy and complications of computed tomography-guided hook wire localization. Ann Thorac Surg..

[10] Mayo JR, Clifton JC, Powell TI, English JC, Evans KG, Yee J (2009). Lung nodules: CT-guided placement of microcoils to direct video-assisted thoracoscopic surgical resection. Radiology.

[11] Lenglinger FX, Schwarz CD, Artmann W (1994). Localization of pulmonary nodules before thoracoscopic surgery: value of percutaneous staining with methylene blue. AJR Am J Roentgenol.

[12] Watanabe K, Nomori H, Ohtsuka T, Kaji M, Naruke T, Suemasu K (2006). Usefulness and complications of computed tomography-guided lipiodol marking for fluoroscopy-assisted thoracoscopic resection of small pulmonary nodules: experience with 174 nodules. J Thorac Cardiovasc Surg.

[13] Chella A, Lucchi M, Ambrogi MC, Menconi G, Melfi FM, Gonfiotti A (2000). A pilot study of the role of TC-99 radionuclide in localization of pulmonary nodular lesions for thoracoscopic resection. Eur J Cardiothorac Surg.

[14] Zaman M, Bilal H, Woo CY, Tang A (2012). In patients undergoing video-assisted thoracoscopic surgery excision, what is the best way to locate a subcentimetre solitary pulmonary nodule in order to achieve successful excision?. Interact Cardiovasc Thorac Surg.

[15] Huang W, Ye H, Wu Y, Xu W, Tang X, Liang Y (2014). Hook wire localization of pulmonary pure ground-glass opacities for video-assisted thoracoscopic surgery. Thorac Cardiovasc Surg.

[16] Li C, Liu B, Jia H, Dong Z, Meng H (2018). Computed tomography-guided hook wire localization facilitates video-assisted thoracoscopic surgery of pulmonary ground-glass nodules. Thorac Cancer.

[17] Zappa C, Mousa SA (2016). Non-small cell lung Cancer: current treatment and future advances. Transl Lung Cancer Res.

[18] Gould MK, Tang T, Liu IL, Lee J, Zheng C, Danforth KN (2015). Recent trends in the identification of incidental pulmonary nodules. Am J Respir Crit Care Med.

[19] Li ZZ, Huang YL, Song HJ, Wang YJ, Huang Y (2018). The value of 18F-FDG-PET/CT in the diagnosis of solitary pulmonary nodules: a meta-analysis. Medicine (Baltimore).

[20] Chen S, Zhou J, Zhang J, Hu H, Luo X, Zhang Y (2011). Video-assisted thoracoscopic solitary pulmonary nodule resection after CT-guided hookwire localization: 43 cases report and literature review. Surg Endosc.

[21] Choi SH, Chae EJ, Kim JE, Kim EY, Oh SY, Hwang HJ (2013). Percutaneous CT-guided aspiration and core biopsy of pulmonary nodules smaller than 1 cm: analysis of outcomes of 305 procedures from a tertiary referral center. AJR Am J Roentgenol.

[22] Fontaine-Delaruelle C, Souquet PJ, Gamondes D, Pradat E, De Leusse A, Ferretti GR (2015). Negative predictive value of transthoracic core-needle biopsy: a multicenter study. Chest..

[23] Nakamura K, Saji H, Nakajima R, Okada M, Asamura H, Shibata T (2010). A phase III randomized trial of lobectomyversus limited resection for small-sized peripheral non-small cell lung cancer (JCOG0802/WJOG4607L). Jpn J Clin Oncol.

[24] Passera E, Rocco G (2017). From full thoracotomy to uniportal video-assisted thoracic surgery: lessons learned. J Vis Surg.

[25] Okada M, Koike T, Higashiyama M, Yamato Y, Kodama K, Tsubota N (2006). Radicalsublobar resection for small-sized non-small cell lung cancer: a multicenter study. J Thorac Cardiovasc Surg.

[26] Shentu Y, Zhang L, Gu H, Mao F, Cai M, Ding Z (2014). A new technique combining virtual simulation and methylene blue staining for the localization of small peripheral pulmonary lesions. BMC Cancer.

[27] Nardini M, Bilancia R, Paul I, Jayakumar S, Papoulidis P, ElSaegh M (2018). (99m) technetium and methylene blue guided pulmonary nodules resections: preliminary British experience. J Thorac Dis.

[28] Watanabe K, Nomori H, Ohtsuka T, Kaji M, Naruke T, Suemasu K (2006). Usefulness and complications of computed tomography-guided lipiodol marking for fluoroscopy-assisted thoracoscopic resection of small pulmonary nodules: experience with 174 nodules. J Thorac Cardiovasc Surg.

[29] Rocco G, Cicalese M, La Manna C, La Rocca A, Martucci N, Salvi R (2011). Ultrasonographic identification of peripheral pulmonary nodules through Uniportal video-assisted thoracic surgery. Ann Thorac Surg.

[30] Ciriaco P, Negri G, Puglisi A, Nicoletti R, Del Maschio A, Zannini P (2004). Video-assisted thoracoscopic surgery for pulmonary nodules: rationale for preoperative computed tomography-guided hookwire localization. Eur J Cardiothorac Surg.

[31] Li W, Wang Y, He X, Li G, Wang S, Xu L, et al. Combination of CT-guided hookwirelocalization and video-assisted thoracoscopic surgery for pulmonarynodular lesions: analysis of 103 patients. Oncol Lett. 4(4):824–828. 10.3892/ol.2012.800 PMC3506590 23205107

[32] Taioli E, Yip R, Olkin I, Wolf A, Nicastri D, Henschke C (2016). Survival after sublobar resection for earlystage lung cancer: methodological obstacles in comparing the efficacy tolobectomy. J Thorac Oncol.

[33] Schuchert MJ, Pettiford BL, Pennathur A, Abbas G, Awais O, Close J (2009). Anatomic segmentectomy for stage I non-small-cell lung cancer: comparison of video-assisted thoracic surgery versus open approach. J Thorac Cardiovasc Surg.

[34] Zhang WH, Bai YY, Guo W, Li M, Chang GX, Liu W, et al. Application of Intrapulmonary Wire Combined With Intrapleural Fibrin Glue in Preoperative Localization of Small Pulmonary Nodules. 2019;98(4):e14029. 10.1097/MD.0000000000014029 PMC6358377 30681559

